# How to Implement a Full Cohort Longitudinal Integrated Clerkship

**DOI:** 10.1111/tct.70422

**Published:** 2026-04-22

**Authors:** Mark Sudlow, Hugh Alberti, Paul Paes

**Affiliations:** ^1^ School of Medicine Newcastle University Newcastle upon Tyne UK

## 1 Background

Longitudinal integrated clerkships (LICs) aim to maximise learning through interleaving of multiple subject areas delivered simultaneously over a prolonged period, in contrast to the sequential design of traditional block rotations (TBRs) [1, 2]. Differing designs of LIC have been developed, including generalist models—based on placements in general practice or smaller hospitals often in rural settings—or parallel‐streaming models where multiple specialties are experienced in a usually weekly recurring timetable [2]. In all designs, the longer period in each subject area allows continuity of relationships with clinical teachers facilitating more individual support for learners and continuity with individual patients promoting more patient‐centred practice [3, 4]. LICs have been shown to benefit students' academic performance, develop students' caring and professional identities, enhance educators' teaching experiences, develop educators' teaching and support patients, leading to a strong practical, theoretical and ethical case for change towards LIC design [5, 6, 7]. In response, many medical schools have introduced LICs but most for only a small proportion of their students [7, 8]. Given the evidence of better outcomes, there is clearly a compelling argument to scale delivery to full cohorts rather than restricting the benefits to a select few.

**FIGURE 1:**
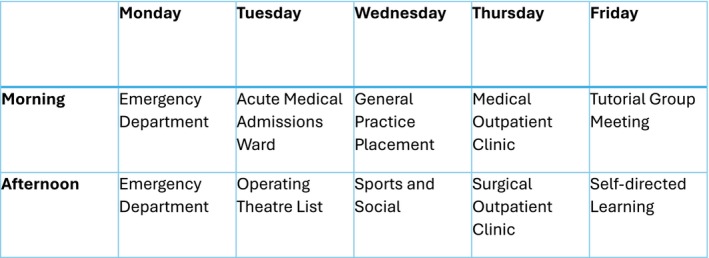
Example weekly student timetable.

**FIGURE 2:**
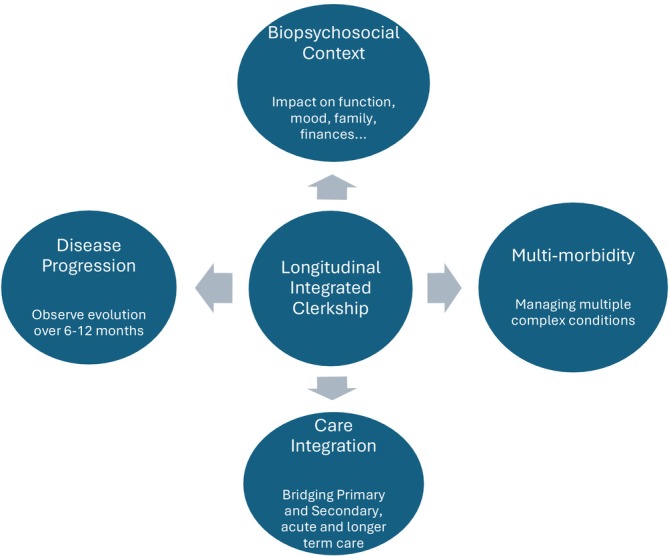
LIC design may be particularly suited to students' learning about long term Conditions.

This paper describes our tips after introducing a whole cohort LIC in a UK medical degree. Curriculum change was motivated by evidence of effectiveness of this delivery model but also by specific content issues we wanted to address. The fourth year in our 5‐year MBBS programme previously contained little clinical placement time. We had identified a deceleration in clinical skills over this period, which we wanted to correct. In expanding clinical placements into Year 4, we wanted to develop enhanced experience in out‐of‐hospital management, particularly of long‐term conditions (Figure 1). Following extensive consultation and preparation, we introduced a full‐cohort (around 350 students) comprehensive parallel‐streaming LIC into Year 4 in 2020–2021, lasting for 8 months and covering all the disciplines of the year: acute and critical care, medicine, elective surgery and general practice (Figure 2). We now have 5 years of experiences, evaluations and results, and what follows are our reflections on the principal lessons learned (see Box 1 for some quotes from faculty and patients [9]).

Box 1 **Why Set Up a Whole Cohort LIC?**

**Teachers**

‘We do not see those patients that often. Whereas, in between consultations, with the student they often give me a good insight. And they [the patient] often disclose a lot of other feelings to them rather than just the medical side of things. So, I certainly felt that the patient was very comfortable with the student and they opened up about how they felt about the investigations arranged and things which they will not have actually said to me in clinic’. Teacher 27.

‘One [student] is needing to have a leave of absence due to family issues and struggles with dyslexia. They want to come back to the surgery over the summer. I suppose I was able to pick up that they were struggling and also be sufficiently challenging with them to make them realise that they were not okay just to continue scraping through because I had that relationship with them. The two others, who were very, very good, I was able to, sort of, pick up on what they did well and stretch them and give them more independence. I think that relationship‐based teaching enables you to recognise when things are not going well, challenge the students appropriately, and stretch them more when they are very good’. Teacher 6.

**Patients**

‘If I had a question about my care they [the student] would get in contact with my GP or the consultant on my case and then they would communicate straight to me … there was no need for me to call the GP and go through all these streets of communication. I could have asked the medical student, “could you get my doctor to send me a message or call me about this?” If they could not provide the answer themselves …‘ Patient 5.

‘You know you are helping someone go forward … she's helped me a great deal, and I think, I feel, I might have helped her understand an older person's worries’. Patient 3.

## 2 Tip 1—Fully Utilise New Placement Opportunities

The LIC design allowed us to utilise large numbers of individual placements in ambulatory, day case, outpatient and community settings, which had not been accessible for the large groups in TBRs. This expanded the number of placements available beyond the already oversaturated and often unsatisfactory inpatient wards and allowed us to deliver real experience in the community management of long‐term conditions, which had been lacking in our previous programme. It also required us to recruit new, more senior and long‐term staff as placement supervisors because, at least in the United Kingdom, resident doctors have minimal presence in these settings or clinical experience to share.

*This expanded the number of placements available beyond the already oversaturated and often unsatisfactory inpatient wards and allowed us to deliver real experience in the community management of long‐term conditions, which had been lacking in our previous programme*.

## 3 Tip 2—Expand and Train Senior Faculty

We needed more teachers to deliver an LIC than a TBR. In our LIC, most teachers have between one and four students on a weekly half‐day placement. Placement supervisors are usually long‐term and more senior staff who can provide continuity over a year, rather than resident doctors who are themselves on relatively short rotations.

Teaching on an LIC seemed intuitive to our general practitioner teachers but alien to our hospital‐based faculty who needed both convincing and training. They were hesitant about their ability to deliver broader outcomes or discuss topics outside of their own specialty and anxious that other students were missing specialty‐specific competencies they previously delivered. It is critical to address these concerns to promote recruitment, ensure effective teaching and avoid undermining of the course from teachers nostalgic for TBR.

We ran events for hospital teachers to explain our view that undergraduate education should predominantly instil skills and attitudes of consultation, reasoning and decision making that are core to all specialties. We aimed to improve teachers' ability to identify and deliver on opportunities to develop these transferrable skills in students. To manage faculty numbers and geographical spread, we used in‐depth events with key educational leaders in each hospital to empower them in their local faculty development and short events on‐line and brief written materials aimed at the broader clinician teacher audience.

## 4 Tip 3—Build Community

Engagement in a clinical or broader community is a feature of most LICs reported in the literature and may improve engagement and provide social support for students [10]. In a whole‐cohort LIC, largely based on outpatient and community placements of individual students with individual senior clinicians, we found that many students paradoxically felt isolated, because they saw other students less frequently and had less shared experiences than when moving in large groups around TBRs.

*In a whole‐cohort LIC, largely based on outpatient and community placements of individual students with individual senior clinicians, we found that many students paradoxically felt isolated, because they saw other students less frequently and had less shared experiences than when moving in large groups*.

To address this, we encouraged teachers to actively integrate their students into the community of their healthcare team. We replaced an asynchronous remote online learning component, which had run for 2 days each week with acute and critical care placements with nearer peer teachers. We arranged fortnightly tutorial groups of 8–10 students to build student communities, share and reflect on clinical experiences and specialist knowledge and derive commonalities between experiences in different settings.

## 5 Tip 4—Align Assessment

A whole‐cohort LIC allowed us to introduce changes to assessment that did not need to match a parallel TBR cohort. TBRs often have assessments of specialty‐specific knowledge at the end of each block, timing that can disadvantage students in small‐cohort LICs who are out of synchrony with their TBR peers. A whole‐cohort LIC is liberated from this pattern and allows realignment of assessment towards skills in consultation, reasoning and decision making that are not speciality specific—skills that are arguably more important to clinicians and patients.

*A whole‐cohort LIC is liberated from this pattern and allows realignment of assessment towards skills in consultation, reasoning and decision making that are not speciality specific*.

We introduced frequent programmatic assessments of these transferable clinical skills based on observations of students in patient consultations spread throughout the academic year. These are cumulatively summative but also provide formative feedback on clinical development. More recall‐based written assessments occur only at the end of the LIC when all students have completed their full experience.

## 6 Tip 5—Enhance Administration and Support

Recruitment of teachers and timetabling of students is a major annual administrative challenge. An LIC replaces students moving in large homogeneous groups between repeating blocks with individual students, each with a bespoke timetable. Inevitably, this multiplies the complexities of timetabling a full cohort. In block systems, scheduling can be arranged within individual departments, but in an LIC, change in any individual department schedule affects all other components of the programme, so greater central coordination is required.

Significant administrative and support requirements continue throughout the year. Student timetables may need to adapt as clinicians' commitments change or when they are on leave. Occasionally, student/placement pairings do not go well and need to be corrected or replaced. Even in ideal placements, some students find negotiating an LIC challenging and need help to organise attendance or identify and make use of opportunities.

Some complex timetabling tasks may be amenable to technological solutions, but there is often a need for personal human interventions to maintain student and faculty engagement whilst negotiating compromise.

## 7 Tip 6—Anticipate ‘Desirable Difficulty’

Learning multiple subjects simultaneously is more difficult and feels less effective to students than learning in blocks. This ‘desirable difficulty’ [11] is part of how interleaving produces better learning and is intrinsic to LICs. ‘Desirable’ refers to the end‐outcome of improved learning; it does not feel ‘desirable’ to students experiencing the difficulty. Anticipating this, we have tried to align students' expectations before and during the course through extensive induction and regular in‐year updates and communications.

A proportion of students feel unsettled by the LIC experience, expressing initial anxieties adapting to the newness of the programme design and preparedness for exams [7]. They can view the lack of clear circumscribed specialist learning as detrimental and experience the challenge of learning in this more effective way as uncomfortable. This proportion is likely to be larger when students are neither selecting nor being selected for the LIC. Occasionally, students may have difficulties that benefit from additional support or a placement change, but this initial anxiety is part of how this curriculum design produces longer term benefit. Be prepared for discontent amongst some, and for the difficult task of distinguishing between those who will benefit from help, and those for whom ‘help’ would paradoxically undermine longer term goals [12].

Course evaluation is likely to reflect this desirable difficulty and needs careful handling at cohort and individual level. Our senior team has been very supportive of our LIC but has undoubtedly received more student concerns about this course than TBRs. Feedback will improve but be prepared in early stages to respond to some negative student feedback in a way that neither belittles students' difficulties nor dilutes the potential positive outcomes that arise from difficulty.

## 8 Conclusion

Many medical schools have introduced small‐scale LICs for selected students. In moving directly to a full‐cohort LIC, we have encountered challenges described in other programmes around identifying and training faculty, the complicated logistics of scheduling and the need to build community [13, 14]. We also found opportunities to expand placements into previously unused clinical areas and to realign assessment particularly valuable to a full cohort. The need to manage negative responses to the challenge of learning on an LIC from unselected students was an unexpected consequence probably amplified by full‐cohort implementation. We hope these reflections assist others in this endeavour: Scaling LICs to whole cohorts will be essential if the benefits are to be realised more broadly and equitably.

*Scaling LICs to whole cohorts will be essential if the benefits are to be realised more broadly and equitably*.

## Author Contributions

**Mark Sudlow:** writing – original draft, writing – review and editing. **Hugh Alberti:** conceptualization, writing – original draft, writing – review and editing. **Paul Paes:** conceptualization, writing – original draft, writing – review and editing.

## Funding

All authors are employed by the Newcastle University. No other funding was received.

## Ethics Statement

The authors have nothing to report.

## Conflicts of Interest

The authors declare no conflicts of interest.

## Data Availability

Data sharing is not applicable to this article, as no datasets were generated or analysed during the current study.
